# A robust method for RNA extraction and purification from a single adult mouse tendon

**DOI:** 10.7717/peerj.4664

**Published:** 2018-04-24

**Authors:** Mor Grinstein, Heather L. Dingwall, Rishita R. Shah, Terence D. Capellini, Jenna L. Galloway

**Affiliations:** 1 Center for Regenerative Medicine, Department of Orthopaedic Surgery, Massachusetts General Hospital, Harvard Medical School, Boston, MA, USA; 2 Department of Human Evolutionary Biology, Harvard University, Cambridge, MA, USA; 3 Broad Institute of Harvard and MIT, Cambridge, MA, USA; 4 Harvard Stem Cell Institute, Cambridge, MA, USA

**Keywords:** Tendon, RNA analysis, Skeletal tissues

## Abstract

**Background:**

Mechanistic understanding of tendon molecular and cellular biology is crucial toward furthering our abilities to design new therapies for tendon and ligament injuries and disease. Recent transcriptomic and epigenomic studies in the field have harnessed the power of mouse genetics to reveal new insights into tendon biology. However, many mouse studies pool tendon tissues or use amplification methods to perform RNA analysis, which can significantly increase the experimental costs and limit the ability to detect changes in expression of low copy transcripts.

**Methods:**

Single Achilles tendons were harvested from uninjured, contralateral injured, and wild type mice between three and five months of age, and RNA was extracted. RNA Integrity Number (RIN) and concentration were determined, and RT-qPCR gene expression analysis was performed.

**Results:**

After testing several RNA extraction approaches on single adult mouse Achilles tendons, we developed a protocol that was successful at obtaining high RIN and sufficient concentrations suitable for RNA analysis. We found that the RNA quality was sensitive to the time between tendon harvest and homogenization, and the RNA quality and concentration was dependent on the duration of homogenization. Using this method, we demonstrate that analysis of *Scx* gene expression in single mouse tendons reduces the biological variation caused by pooling tendons from multiple mice. We also show successful use of this approach to analyze *Sox9* and *Col1a2* gene expression changes in injured compared with uninjured control tendons.

**Discussion:**

Our work presents a robust, cost-effective, and straightforward method to extract high quality RNA from a single adult mouse Achilles tendon at sufficient amounts for RT-qPCR as well as RNA-seq. We show this can reduce variation and decrease the overall costs associated with experiments. This approach can also be applied to other skeletal tissues, as well as precious human samples.

## Introduction

Tendon injuries are common problems for active individuals and the aging population (Kaux et al., 2011). Treatment options include physical therapy and surgical intervention, but pain and limited mobility often persist, making complete restoration of tendon function challenging (Nourissat, Berenbaum & Duprez, 2015). Our current understanding of the molecular and cellular pathways regulating tendons during homeostasis, healing, and aging are limited. Several studies using large animal models such as sheep, rabbits, and rats have provided important information about tendon injury, biomechanics, surgical techniques, and bioengineering strategies for tendon repair (Voleti, Buckley & Soslowsky, 2012). Other studies have used mouse genetics to gain an understanding of the molecular and cellular response of tendons to acute injuries, changing load environments, and in gene loss-of-function models (Dunkman et al., 2014; Dyment et al., 2014; Howell et al., 2017; Mendias, Bakhurin & Faulkner, 2008; Wang et al., 2017). The mouse system offers unique advantages for implementing mechanistic studies of tendon biology as they permit genetic lineage tracing and conditional knockout strategies, and they can be housed simply and in large numbers to improve sample sizes for functional studies. Even with inbred mouse strains, inter-animal variation can affect the conclusions drawn from gene expression analyses (Sultan et al., 2007; Watkins-Chow & Pavan, 2008). Therefore, the use of several biological replicates of tendon tissues obtained from individual mice for RNA analysis is essential for furthering our mechanistic understanding of tendon biology.

Mature tendons are comprised of type I collagen, which is arranged in a highly ordered hierarchical manner along the long axis of the tissue (Kannus, 2000). Tendon cells lie between these organized fibrils and are surrounded by a hydrophilic, glycoprotein-rich ground substance (Bi et al., 2007; Kannus, 2000; Yoon & Halper, 2005). This dense, fibrous, water-rich matrix that surrounds the tendon cells poses a significant challenge for the acquisition of high-quality RNA. In addition, tendons have low cell density compared with other tissues such as muscle or liver, resulting in minimal RNA yield per gram of tissue (Kannus, 2000; Reno et al., 1997).

Previous studies have described protocols for RNA extraction from human or larger mammalian animal models such as rabbit (Ireland & Ott, 2000; Reno et al., 1997), but analyzing RNA from small animal models such as mouse can be more difficult. This issue has led to several different strategies for achieving RNA yield and quality sufficient for gene expression analysis by RT-qPCR or RNA-seq. RNA amplification methods have permitted gene expression analysis of single injured and uninjured tendons (Dunkman et al., 2014), but this can be prohibitively expensive for analyzing a large number of samples or target genes. In addition, studies in other tissues have shown that global pre-amplification can lead to biased results and increased false negative rates, especially for low- and medium-copy transcripts (Dunkman et al., 2014). Targeted pre-amplification methods have been developed to minimize PCR bias by using multiplexed primer pools at low concentrations combined with few PCR cycles (Jang et al., 2011). The resulting amplified cDNA can be used either in SYBR Green-based or probe-based qPCR assays. While such amplification methods have been shown to be highly sensitive and yield less-variable RT-qPCR results compared to global cDNA amplification (Kroneis et al., 2017), the complex nature of the PCR amplification reactions imposes some limitations. First, targeted pre-amplification reactions require precision to yield usable cDNA. Because input sample concentrations, mRNA copy number, PCR cycle number, the specific combination of targets, and primer pool concentration can all affect the success of targeted pre-amplification, each assay must be individually optimized (Korenková et al., 2015; Kroneis et al., 2017). Improperly formulated reactions can lead to poor specificity and sensitivity of downstream qPCR, especially when using SYBR Green chemistry (Andersson et al., 2015). Such optimizations are time consuming and are not generalizable to different samples and different gene sets. Additionally, the nature of amplifying a specific set of targets inherently limits the possible downstream use of the pre-amplified samples.

Mendias and colleagues and Nielson and colleagues have performed gene expression analysis on a single mouse Achilles or plantaris tendon in different loss-of-function mouse models or in altered loading conditions (Mendias, Bakhurin & Faulkner, 2008; Mendias et al., 2012; Nielsen et al., 2014). This approach is not widespread in the literature and these studies do not report on the RNA integrity, although they do report sample purity (260/280 ratio). However, there are examples of many studies that pool a large number of tendons (e.g., 12–20 individual tendons) (Bell et al., 2013; Trella et al., 2017). Not only does this increase the mouse cohort size and experimental costs, but it can also inflate the inter-sample variation, which may explain some of the large variability in transcript abundance that was found in subsets of their gene expression analysis (Trella et al., 2017). Lastly, other studies have focused on tendon-derived cell populations such as tendon stem/progenitor cells (Bi et al., 2007). This approach results in robust RNA yields, but it queries a cell population that has been expanded in culture and could have altered transcriptomic and epigenomic states compared with that of native tendon tissue.

The various technical limitations associated with obtaining high-quality, high-yield RNA using existing protocols enlarges the cohorts of mice needed for statistical analysis, and can hinder the use of RT-qPCR or functional genomic assays such as RNA-seq on single adult mouse tendons. Here, we present a robust, low-cost, and straightforward RNA isolation protocol that enables the isolation of high-integrity RNA from a single mouse Achilles tendon. We show that pooling tendon samples inflates estimates of biological variance for gene expression data in RT-qPCR analysis. We apply this method to analysis of injured and contralateral uninjured tendons to demonstrate the detection of significant and reproducible gene expression changes. In addition, this method can be used to purify high quality RNA from other musculoskeletal tissues, making it easily adaptable to multiple connective and skeletal tissue types, or from difficult to obtain tissues from humans or other organisms.

## Methods

### Mouse studies

Achilles tendons were collected from wild type C57BL/6J mice between three to five months of age (Jackson Laboratories 00664, *n* = 30 total). To compare gene expression levels between injured and uninjured Achilles tendons in the same mouse, excisional Achilles tendon injuries were performed using a 0.3 mm biopsy punch as described (Beason et al., 2012). The incision was closed with 6-0 Ethilon nylon sutures and the tendons were harvested 30 days after injury for analysis. Mice were housed, maintained, and euthanized according to American Veterinary Medical Association guidelines. All experiments were performed according to our Massachusetts General Hospital Institutional Animal Care and Use Committee (IACUC: 2013N000062) approved protocol.

### RNA extraction and purification

Dissected Achilles tendons were placed immediately into 1.5 ml tubes containing 500 μl of TRIzol reagent (15596026; Invitrogen, Carlsbad, CA, USA) and high impact zirconium 1.5 mm beads (30–40 beads per tube, D1032-15, Benchmark, Sayreville, NJ, USA). Samples were homogenized in two 180-s rounds of bead beating at 50 Hz (BeadBug microtube homogenizer; Benchmark Scientific, Sayreville, NJ, USA). Samples were then moved directly to dry ice or −80 °C for longer storage up to six months.

Alternative tissue disruption procedures that were tested included homogenization of both fresh and frozen tendons in 500 μl TRIzol with a Polytron handheld homogenizer (PT 1200E, Kinematica AG, Luzern, Switzerland) until tissue was visibly disrupted (60–90 s). Cryogrinding of samples was tested using a freezer mill (SPEX 6875). Achilles tendons were snap frozen in liquid nitrogen and transferred to a super-cooled SPEX grinding cylinder (SPEX 6751C4; Metuchen, NJ, USA) and pulverized in a bath of liquid nitrogen for 3 min. Ground samples were collected by rinsing the cylinder with 500 μl TRIzol and transferred to a 1.5 ml tube. For enzymatic digestion, tendons were placed in 2 ml Eppendorf tubes with 1 ml digestion solution containing 0.2% collagenase II (Worthington, LS004176, Lakewood, NJ, USA) in DMEM (11965; Gibco, Gaithersburg, MD, USA) containing 0.1% Penicillin/Streptomycin (30002cl; Corning, NY, USA) and 1% Hepes (15630-080; Gibco, Gaithersburg, MD, USA). Tubes were kept on ice during the dissection period and were incubated together in a 37 °C shaking water bath for 90 min. In order to digest remaining matrix, we added 200 μl of 0.2% collagenase I (17100-017; Gibco, Gaithersburg, MD, USA) and 300 μl of 0.4% Dispase (17105-041; Gibco, Gaithersburg, MD, USA) to the partially digested samples and incubated at 37 °C for an additional 30 min. Following the digestion, the samples were centrifuged at 500 RCF (*g*) for 5 min, the supernatant was aspirated, and 500 μl TRIzol was added. All homogenized samples were stored at −80 °C until RNA isolation.

To extract RNA, the samples were thawed on ice followed by a 5 min incubation at room temperature. Samples were quickly spun in the sample tubes and the homogenate was moved to a new Eppendorf tube, leaving behind the beads and residual tissue. Next, a chloroform extraction was performed using double the recommended ratio of chloroform to TRIzol, which has been shown to increase RNA yield in small samples (Macedo & Ferreira, 2014). One hundred microliters of chloroform was added to the homogenate and vortexed well for approximately 1 min. The TRIzol/chloroform mixture was then moved to a 1.5 ml MaXtract high density tube (129046; Qiagnen, Germantown, MD, USA), incubated at room temperature for 2–3 min, and spun ≥12,000 RCF at 4 °C for 15 min. MaXtract tubes contain a sterile gel that forms a barrier between the RNA-containing aqueous phase and the TRIzol/chloroform upon centrifugation at 4 °C, thus minimizing carryover of organic solvents leading to an overall reduction in sample contamination. After centrifugation, the aqueous phase was transferred to a clean 1.5 ml Eppendorf tube and an equal volume of 100% ethanol was added to the aqueous phase and mixed well. At this stage, the RNA/ethanol mix was typically stored at −80 °C. We have found that brief incubation of this mixture at −80 °C improved the total RNA yield, but it is not required.

RNA purification was next performed using the ZR Tissue & Insect RNA MicroPrep kit (R2030; Zymo Research, Irvine, CA, USA) or the Direct-Zol systems (R2050, R2060; Zymo Research, Irvine, CA, USA). Based on typical tendon yields, the ZymoSpin IC spin columns are optimal for use with RNA extracted from single tendons as these columns can purify up to 5 μg of RNA in as little as 6 μl eluate. However, this protocol also has been successfully used with ZymoSpin IIC columns, which require a larger elution volume. After adding the RNA/ethanol mix to the spin column, the standard Zymo purification protocol was used with the following modifications. First, a 15 min on-column DNase I treatment was added to minimize genomic DNA contamination. An extra wash step was included to improve sample purity. Prior to elution, columns were spun for an additional 2 min at maximum speed to remove residual ethanol. RNA was eluted in 15 μl RNase/DNase free water that was pre-warmed to 55–60 °C to maximize the RNA recovery from the spin column. RNA concentration was measured via fluorometric quantitation (Qubit HS RNA assay; Q32852; Invitrogen, Carlsbad, CA, USA) and sample quality was determined by spectrophotometric analysis (NanoDrop 2000c; ThermoFisher Scientific, Waltham, MA, USA) as well as capillary electrophoresis (2100 Bioanalyzer; Agilent, Santa Clara, CA, USA). The final RNA product was stored at −80 °C for RT-qPCR analysis.

### RT-qPCR, data analysis, and statistics

One hundred ng total RNA was reverse transcribed with oligo(dT)_20_ primers using the SuperScript IV First Strand Synthesis System (ThermoFisher Scientific, 18091050) and a no-reverse transcriptase control was included for every sample. A total of 2 ng cDNA template was amplified for 40 cycles in each SYBR green qPCR assay (Applied Biosystems, 4367659, Waltham, MA, USA) using a final primer concentration of 200 nM. All assays were performed in technical triplicate using either a LightCyclerII 480 (pooled samples; Roche, Penzberg, Germany) or a StepOnePlus Real Time PCR system (injury samples; Applied Biosystems, Foster City, CA, USA). Three independent biological replicates were run per condition for both sets of RT-qPCR. *Gapdh* was used as the reference gene for all samples (see Table 1 for primer sequences).

**Table 1:** RT-qPCR primer sequences.

Gene	Forward primer (5′–3′)	Reverse primer (5′–3′)
*Sox9*	AGTACCCGCATCTGCACAAC	TACTTGTAATCGGGGTGGTCT
*Col1a2*	CCAGCGAAGAACTCATACAGC	GGACACCCCTTCTACGTTGT
*Scx*	AAGTTGAGCAAAGACCGTGAC	AGTGGCATCCACCTTCACTA
*Gapdh*	TGTTCCTACCCCCAATGTGT	GGTCCTCAGTGTAGCCCAAG

All analyses were conducted in R 3.4.3 (R Core Team, 2017). For the pooling experiment, summary statistics were calculated for *Scleraxis* (*Scx*) and *Gapdh* technical and biological replicate cycle threshold (*C*_T_) values independently. Variance estimates for *Scx* Δ*C*_T_ relative expression were calculated using standard error propagation techniques. Relative expression values for *Collagen Ia2* (*Col1a2*) and *SRY-Box9* (*Sox9*) were calculated for the injury analysis using the ΔΔ*C*_T_ method (Livak & Schmittgen, 2001) and injury samples were normalized to their corresponding uninjured contralateral controls. Statistical differences between injured and uninjured samples from three biological replicates (*n* = 3 mice) were analyzed via Welch’s *t*-test (Welch, 1947) on the Δ*C*_T_ values.

## Results

Several tissue disruption methods were tested in order to achieve optimal RNA quality and quantity from a single mouse tendon. Among those tested were enzymatic digestion, cryogenic grinding (manual and mill), shearing with a handheld homogenizer (i.e., rotor–stator), and bead beating. Capillary electrophoresis was performed on purified RNA using a Bioanalyzer RNA Nano chip (Agilent, Santa Clara, CA, USA). RNA integrity number (RIN), a quantification of degradation, was calculated by the accompanying Agilent software based on the electropherogram for a given sample; a RIN of ten indicates completely intact RNA whereas a RIN of one indicates severely degraded RNA. Enzymatic digestion produced intact RNA (RIN > 7), but low RNA yield (≤1 ng/μl). Cryogenic grinding and handheld homogenizer dissociation methods resulted in low yield (≤5 ng/μl) and poor RNA purity and integrity (RIN ≤ 3) (Table 2A). Bead beater homogenization was found to produce the best results in terms of RNA quality (i.e., RIN ≥ 6.5) and quantity (≥50 ng/μl), and minimized carryover between samples. Additionally, bead beating was easily combined with standard TRIzol extraction and commercially available purification methods.

**Table 2:** Purity measurements for different homogenization methods and single versus pooled tendon samples.

(A) RNA purity (260/280 ratio), concentration, and RIN measurements are poor following Polytron or Spex freezer mill homogenization methods						
Sample	Number of tendons	Conc. (ng/μl)	Homogenization method	RIN	260/280	260/230
8wk poly 1	One	32.5	Polytron	None	1.57	0.49
8wk poly 2	One	51	Polytron	2.5	1.62	0.34
8wk poly 3	Three	71.2	Polytron	2.6	1.58	0.33
8wk poly 4	Three	29.4	Polytron	None	1.56	0.65
8wk spex 1	Four	170	Spex freezer mill	2.4	1.44	0.22
8wk spex 2	Four	80	Spex freezer mill	2.4	1.47	0.25
8wk spex 3	One	4.4	Spex freezer mill	2.3	1.44	0.31
8wk spex 4	One	4.7	Spex freezer mill	None	2.26	0.01

(B) Purity measurements are high regardless of pooling level with our bead beating protocol						
Sample	Number of tendons				260/280	260/230
1AT_1	One				2.06	1.97
1AT_2	One				2.04	2.06
1AT_3	One				2.01	2.03
2AT_1	Two				2.04	1.91
2AT_2	Two				1.99	1.88
2AT_3	Two				2.05	2.02
4AT_1	Four				2.06	2.05
4AT_2	Four				2.05	2.07
4AT_3	Four				2.05	2.06
6AT_1	Six				2.06	2.05
6AT_2	Six				2.06	2.05
6AT_3	Six				2.05	2.05
8AT_1	Eight				2.07	2.08
8AT_2	Eight				2.05	2.06
8AT_3	Eight				2.06	2.05

**Figure 1:**
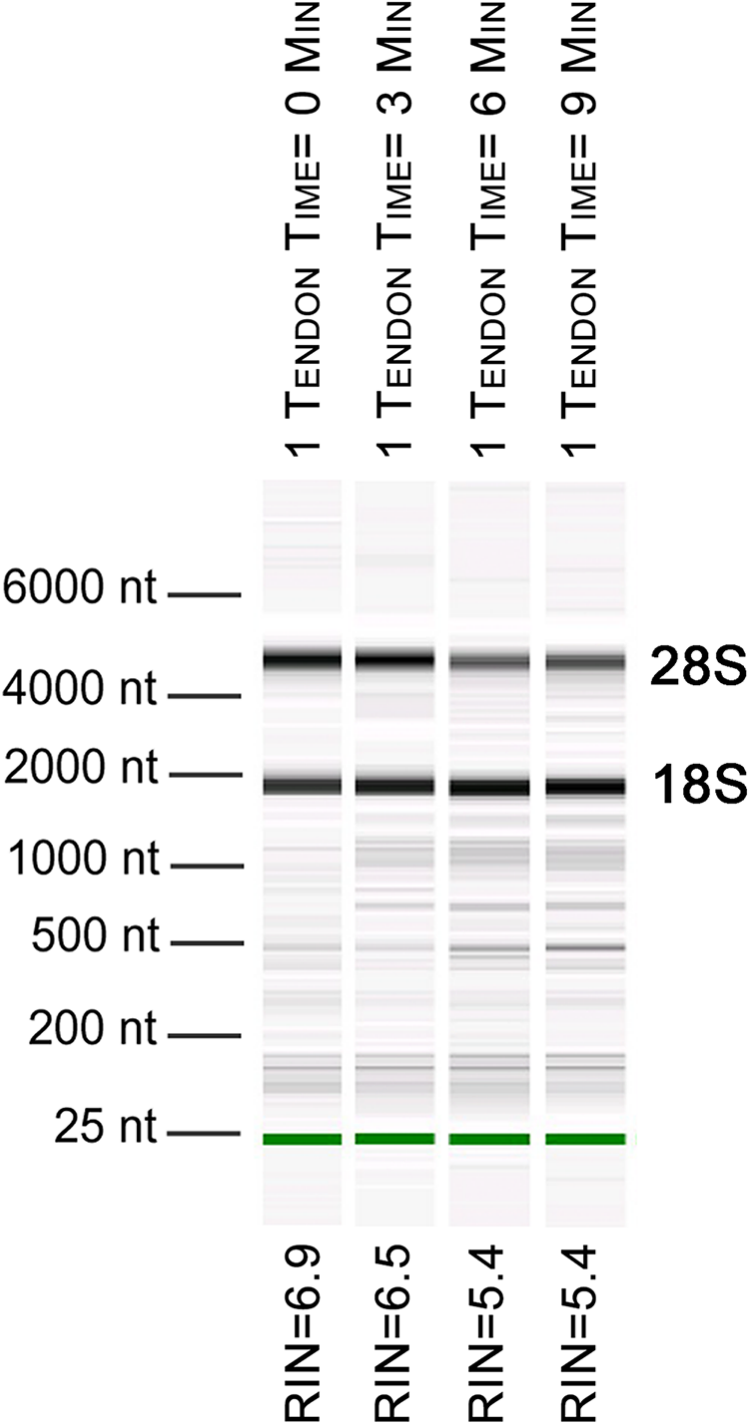
Length of time between dissection and processing affects RNA integrity. Electropherogram digital gel via Bioanalyzer shows integrity of RNA isolated from single Achilles tendons that were kept on ice for various lengths of time (0, 3, 6, 9 min) before homogenization. All were homogenized for 360 s. Longer wait times prior to homogenization reduce RNA quality. 18S and 28S are indicated and the green band is a marker.

**Figure 2:**
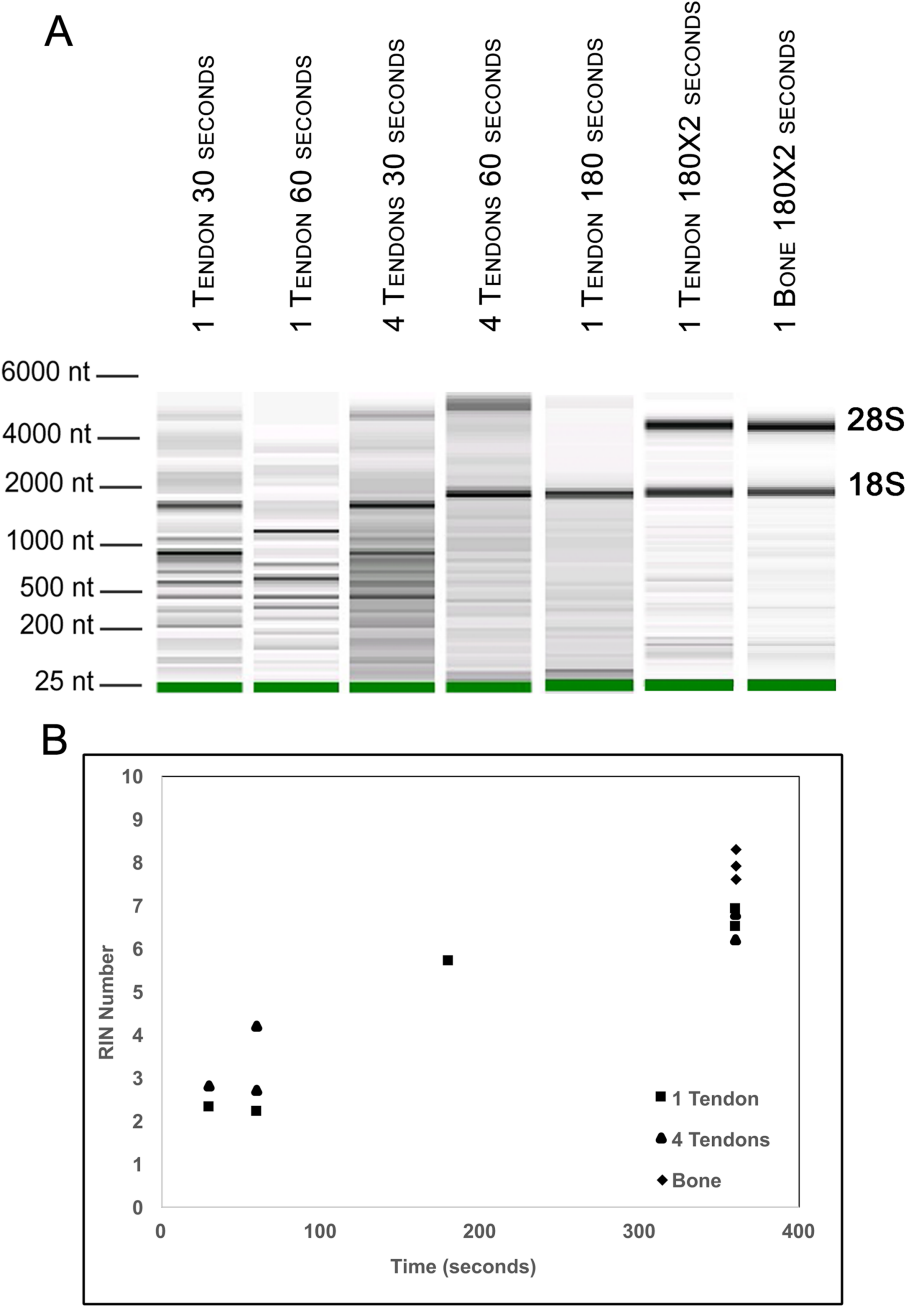
Optimization of homogenization regime. Single Achilles tendons and pools of four tendons were subjected to different durations of bead beating homogenization: 30, 60, 180, and 360 s (in two rounds of 180 s). The electropherogram digital gel shows that the longest beating time resulted in the most intact RNA, as evidenced by the strong 28s and 18s bands with 360 s (A). RIN values called by Agilent software also show the improvement in quality with longer beating time (B).

To further evaluate our bead beating homogenization method, we performed additional experiments examining the level of degradation that occurs prior to homogenization as well as during homogenization. To address the former, single Achilles tendons from similarly aged mice were left in sterile 1× PBS on ice following dissection for up to 9 min before homogenization to simulate waiting times involved in batch dissection. The shortest time between dissection and homogenization (0–30 s) yielded more intact RNA (RIN = 6.5) while longer wait times resulted in more degraded RNA (9 min processing time RIN = 5.4; Fig. 1). This demonstrates that measurable degradation can occur prior to sample homogenization, and occurs with increases in time after dissection on the order of only minutes (Fig. 1). Therefore, processing the tendon(s) immediately following dissection is essential for preserving RNA integrity. We next tested how the duration of bead beating affects RNA quality by varying homogenization times of single and four pooled Achilles tendons. Samples were homogenized for 30, 60, 180, or 360 s (in two consecutive rounds of 180 s; Figs. 2A and 2B). RNA from samples homogenized for less than 60 s suffered more degradation than those that underwent longer homogenization times (Fig. 2B), indicating incomplete homogenization of the tissue during the shorter bead-beating periods. Homogenization times of 720 s did not improve RIN numbers (RIN = 6). This could be due to prolonging the amount of time until RNA extraction or elevation of the temperature with longer homogenization periods, leading to degradation. The temperature of TRIzol is a likely factor as other studies with similar homogenization techniques used methods to lower its temperature (Leite, Magan & Medina, 2012).

**Figure 3:**
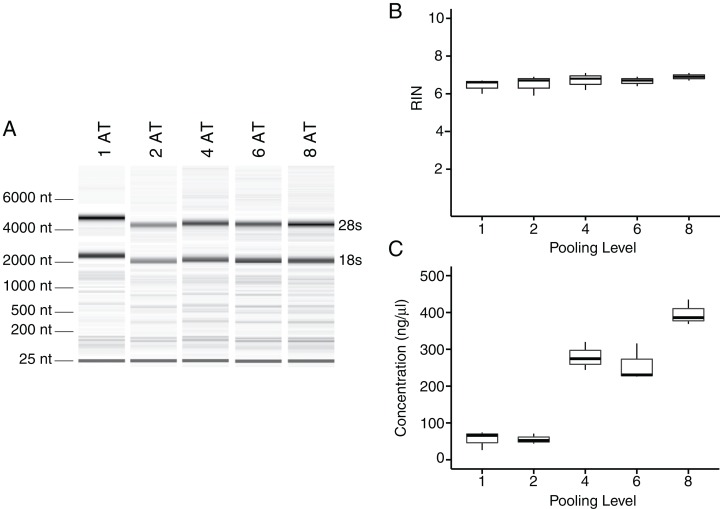
Tendon pooling affects RNA yield but not quality. Representative electropherogram digital gel of RNA from different-sized pools of Achilles tendons demonstrates high integrity RNA across all samples (A). Called RINs for pools (*n* = 3 per pool) demonstrates that RNA quality from a single tendon is comparable to that from pools of tendons. Sample RINs are sufficiently high for use in RNA-seq gene counting and differential expression analysis for as low as one Achilles tendon (B). Concentration of RNA from single or pooled tendons increases with tendon number (*n* = 3 per pool) (C). The middle line represents the median, the box is quartiles 2 and 3 interquartile range (IQR), and whiskers are 1.5× IQR (B, C).

**Figure 4:**
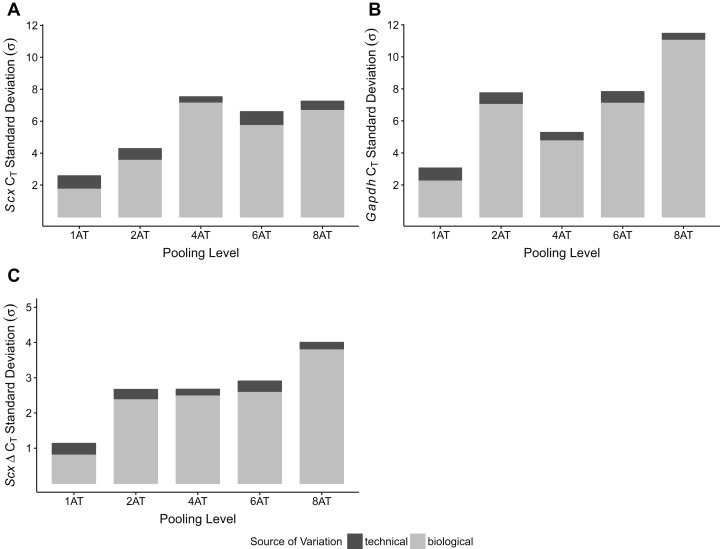
Sample pooling affects estimates of sample variance in RT-qPCR. *C*_T_ standard deviations for *Scx* (A) and *Gapdh* (B) measurements were calculated for the technical replicates (*n* = 3 repeat measurements; dark gray) and for biological replicates (*n* = 3 independent samples; light gray), separately. Δ*C*_T_ was calculated by normalizing *Scx C*_T_ values to *Gapdh*. Technical (dark gray) and biological (light gray) variance estimates were calculated separately (C). All measures (A–C) show that biological variance increases as number of individuals contributing to a pool increases.

To test whether pooling tendons from multiple individuals into one sample prior to homogenization influences RNA integrity, we measured RNA quality from single Achilles tendons as well as pools of differing sizes (two, four, six, and eight tendons, *n* = 3 biological replicates per pooling level; Figs. 3A and 3B). Electropherograms and RIN measurements show that RNA from all pooling levels suffer levels of degradation similar to single Achilles samples (Figs. 3A and 3B). Purity measurements were also similar among single and pooled samples (Table 2B). Therefore, pooling tendons from multiple individuals is not protective against RNA degradation; the only measure that improved with increased pool size was RNA yield (Fig. 3C). To determine if pooling multiple samples affects gene expression measurements, we evaluated gene expression in single and differentially pooled tendon samples described above (*n* = 3 per pooling level) via RT-qPCR. Although we find no gain in RNA quality from pooling, treating pools of tendons from multiple individuals as single biological replicates results in larger standard deviations in *C*_T_ measurements in assays for *Scx* and *Gapdh* (Fig. 4). This leads to larger sample variance estimates for larger pools, driven by differences in Δ*C*_T_ between biological replicates within a group, which impedes the detection of small gene expression changes. Such increases in variance for pooled versus individual samples have also been reported for RNA-seq datasets (Rajkumar et al., 2015).

**Figure 5:**
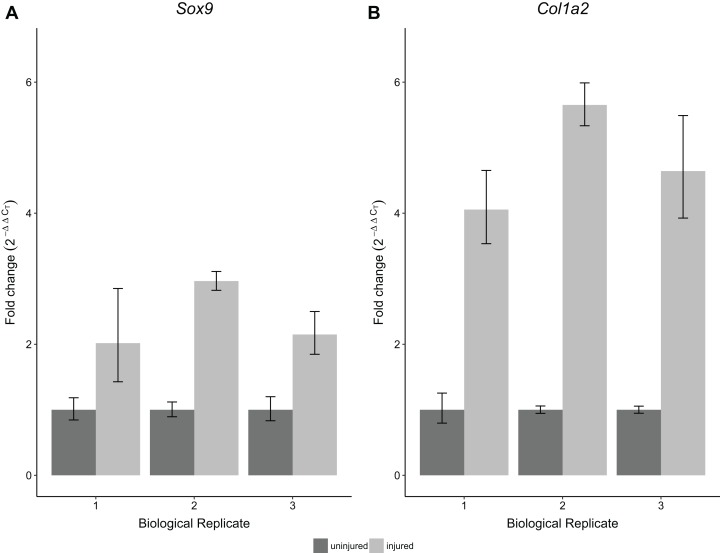
Sensitivity and reproducibility of RT-qPCR on single tendon RNA. RT-qPCR of *Sox9* (A) and *Col1a2* (B) expression of injured Achilles tendons relative to the contralateral tendon of the same mouse at 30 days post-injury. A Welch’s *t*-test shows that both *Sox9* and *Col1a2* expression was significantly different in the injured condition compared to the control tendons (*n* = 3 biological replicates, *p* < 0.05 for *Sox9* and *p* < 0.01 for *Col1a2*).

To validate the performance of the RNA obtained using this protocol, we performed RT-qPCR for *Sox9* and *Col1a2* expression on single Achilles tendons at 30 days following an acute excision Achilles tendon injury. All samples were obtained from single injured and contralateral uninjured Achilles tendons from the same mouse. Using this protocol, we found significantly increased expression of *Sox9* and *Col1a2* in injured Achilles tendons compared with their uninjured contralateral counterparts (*p* < 0.05 for *Sox9* and *p* < 0.01 for *Col1a2*; Fig. 5). These results are consistent with previous studies showing increased expression of *Sox9* and *Col1a2* following tendon injury (Guerquin et al., 2013; Zhang & Wang, 2013), thereby demonstrating the robustness of our method in detecting gene expression changes in single tendon samples.

## Discussion and Conclusion

Obtaining high quality RNA from tendons can be challenging, and this can limit the direction and scope of studies focused on analyzing adult mouse tendon tissues. Whereas a few studies have used single tendons without amplification, many other studies have used amplification or pooling of samples from more than 12 individuals to detect gene expression changes. Both approaches can be expensive due to the high costs associated with amplification kits for multi-gene analysis or the number of mice used for one biological replicate. Dissociation, followed by culture and expansion of tendon-derived cells can yield greater RNA concentrations of high quality, but such approaches cannot be used to study gene expression changes after injury. The approach we describe above provides a straightforward method to consistently obtain high yields of RNA from one Achilles tendon of sufficient quality to perform RT-qPCR analysis without amplification. Our reported RIN scores are also acceptable for standard RNA-seq differential expression analysis. In addition, studies have shown minimal differences between polyA-selected samples of high (RIN > 7) and moderate (RIN = 6–7) RNA integrity, as well as efficacy in correcting for variation in RNA integrity in the differential expression analysis (Jaffe et al., 2017; Romero et al., 2014). Although high RIN values should be the goal, there are some options for computationally managing the effects of moderate RIN, which can prove helpful for studies in which there are limitations in sample quality. However, it must be noted that each RNA-seq library preparation system has specific input RNA requirements, and researchers should ensure that their RNA samples meet all manufacturer qualifications prior to use in a sequencing study.

Our analysis also uncovered key steps that are integral toward generating relatively intact, high yield RNA from the single tendon samples. In particular, we find that the time from dissection to homogenization and storage can significantly impact the quality of the RNA, causing measurable degradation. In this regard, even small delays on the order of minutes could affect overall RNA quality, which could greatly affect differential gene expression analysis. In addition, the duration of homogenization is important for maximizing RNA yield and quality. Homogenization times that are too short or long can result in dramatically different RIN and concentrations regardless of the level of sample pooling.

Similar to previous RNA-seq studies, our RT-qPCR analysis of single and pooled tendon samples revealed that pooling increases the variance of gene expression measurements (Rajkumar et al., 2015). It has been argued that pooling samples from multiple individuals into single biological replicates results in biological averaging and is therefore an appropriate, and even useful, practice in gene expression studies via microarray (Kendziorski et al., 2005). However, genes that are lowly expressed or exhibit subtle differences between conditions would require a larger sample size of pools to achieve adequate statistical power, which would further inflate mouse and reagent cost for RT-qPCR, microarray, or RNA-seq analyses (Shih et al., 2004). This study also highlights the problem of performing RT-qPCR comparisons on a single pool per group (run in technical triplicate), under the assumption that the within-sample variation is representative of the biological variation among all animals of that group. Variance calculated from technical replicates does not estimate biological variance within each group, and is not an appropriate practice. Technical variation arises from noise due to measurement error and therefore is unrelated to biological variation (Kitchen, Kubista & Tichopad, 2010; Vaux, Fidler & Cumming, 2012), necessitating the use of multiple pools for any statistical analysis.

Our tendon RNA extraction method is a robust protocol for obtaining high quality RNA for gene expression assays. It decreases the number of mice required for analysis and avoids extra amplification steps, making it straightforward, cost-effective, and easily accessible to researchers new to the tendon field. By providing a means for reproducibly analyzing one Achilles tendon, this method also reduces measurement error associated with pooling tendons from multiple individuals. Moreover, our protocol permits the use of internal comparisons between a limb that has undergone experimental manipulation (e.g., injury or unloading) and the contralateral control limb within the same animal. In addition to facilitating larger-scale RT-qPCR studies, we believe this method will make highly dimensional gene expression analysis, such as RNA-seq, accessible to more researchers studying musculoskeletal tissues, thus opening new frontiers in tendon biology.

## Supplemental Information

10.7717/peerj.4664/supp-1 Supplemental Information 1 R script for Pooling variance calculations.

10.7717/peerj.4664/supp-2 Supplemental Information 2 R script for injury expression analysis.

10.7717/peerj.4664/supp-3 Supplemental Information 3 Raw data from Bioanalyzer.

10.7717/peerj.4664/supp-4 Supplemental Information 4 Raw data from BioAnalyzer and Qubit.

10.7717/peerj.4664/supp-5 Supplemental Information 5 Ct values from RT-qPCR for mouse tendon injury in Fig. 5.

10.7717/peerj.4664/supp-6 Supplemental Information 6 Ct values from RT-qPCR for Fig. 4.

## Additional Information and Declarations

### Data Availability

The following information was supplied regarding data availability: The data and R scripts are provided as Supplemental Dataset Files.
