# Supplementary figures and images for: A robust method for RNA extraction and purification from a single adult mouse tendon
Source: PeerJ. 2018 Apr 24;6:e4664. doi: 10.7717/peerj.4664 (PMC5922231; doi:10.7717/peerj.4664)

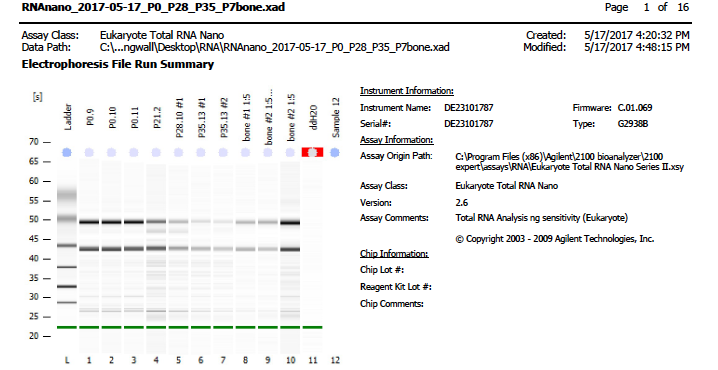


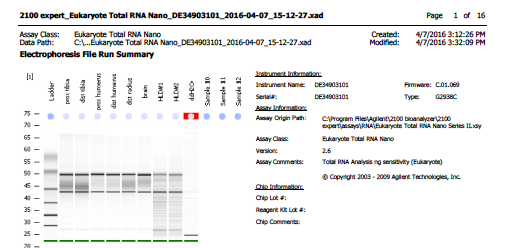


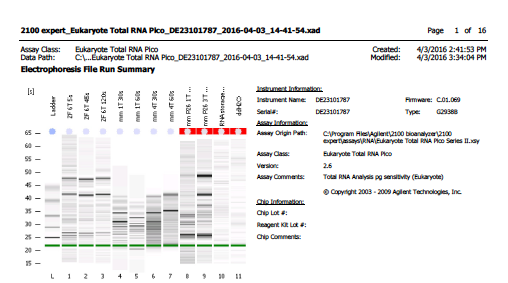


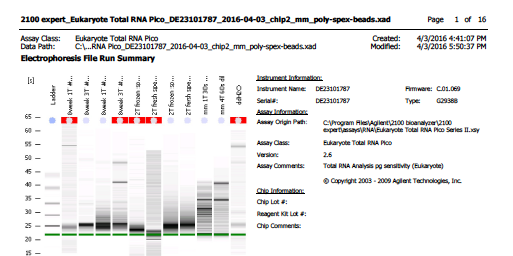


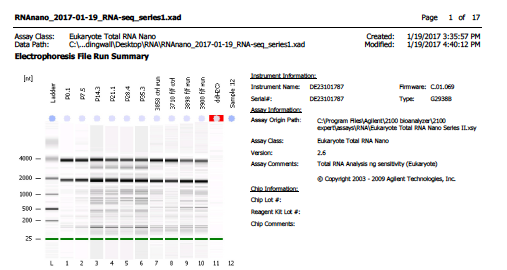


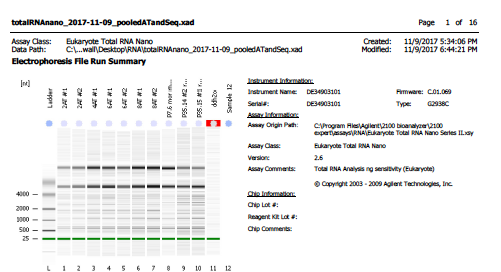


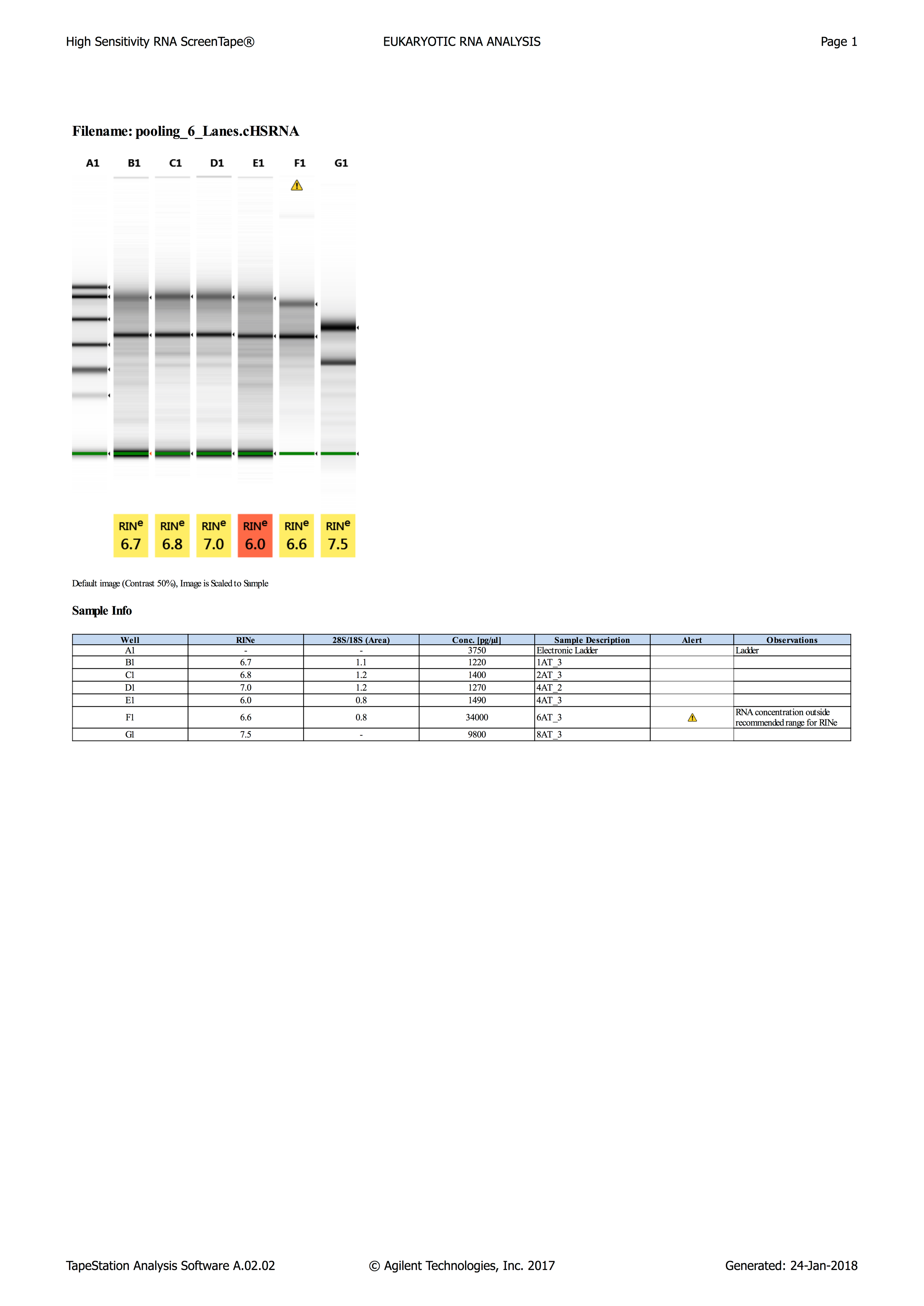


*run on Agilent TapeStation HighSensitivity RNA screen tape

Supplement: Supplemental Information 3 [file peerj-06-4664-s003.docx]
