# Supplementary material for: A robust method for RNA extraction and purification from a single adult mouse tendon
Source: PeerJ. 2018 Apr 24;6:e4664. doi: 10.7717/peerj.4664 (PMC5922231; doi:10.7717/peerj.4664)
Supplement: Supplemental Information 6 [file peerj-06-4664-s006.rtf]

J8	Gapdh	Control	5mo_2	1AT	1	18.53	TargetK8	Gapdh	Control	5mo_2	1AT	2	18.27	TargetL8	Gapdh	Control	5mo_2	1AT	3	18.24	TargetA8	Scx	           Target	           5mo_2	1AT	1	24.91	TargetB8	Scx	           Target	           5mo_2	1AT	2	25.14	TargetC8	Scx	           Target	           5mo_2	1AT	3	24.84	TargetJ7	Gapdh	Control	5mo_1	1AT	1	18.65	TargetK7	Gapdh	Control	5mo_1	1AT	2	18.25	TargetL7	Gapdh	Control	5mo_1	1AT	3	18.76	TargetA7	Scx	Target	5mo_1	1AT	           1         	 25.14	TargetB7	Scx	Target	5mo_1	1AT	           2 	            25.17	TargetC7	Scx	Target	5mo_1	1AT	           3	            25.28	TargetA9	Scx	Target	5mo_3	1AT	            1	            24.53	TargetB9	Scx	Target	5mo_3	1AT	            2	            23.77	TargetC9	Scx	Target	5mo_3	1AT	            3	            23.87	TargetJ9	Gapdh	Control	5mo_3    	1AT	1	17.48	TargetK9	Gapdh	Control	5mo_3	1AT	2	16.98	TargetL9	Gapdh	Control	5mo_3	1AT	3	16.93	TargetJ10	Gapdh	Control	2AT_1	2AT	    1	            22.69	TargetK10	Gapdh	Control	2AT_1	2AT	    2	            22.6	TargetL10	Gapdh	Control	2AT_1	2AT	    3	            22.38	TargetA10	Scx	             Target	2AT_1	2AT	    1	            24.15	TargetB10	Scx	            Target	2AT_1	2AT	    2	            23.84	TargetC10	Scx	            Target	2AT_1	2AT	    3	            23.87	TargetJ11	Gapdh	Control	2AT_2	2AT	1          	18.91	TargetK11	Gapdh	Control	2AT_2	2AT	2	           18.81	TargetL11	Gapdh	Control	2AT_2	2AT	3	           18.77	TargetA11	Scx	Target	                     2AT_2	2AT     1	            25.46	TargetB11	Scx	Target	                     2AT_2	2AT     2                    24.94	TargetC11	Scx	Target	                    2AT_2	2AT	 3	           24.94	TargetJ12	Gapdh	Control	2AT_3	2AT	1	           17.84	TargetK12	Gapdh	Control	2AT_3	2AT	2	           18.52	TargetL12	Gapdh	Control	2AT_3	2AT	3	           18.15	TargetA12	Scx	          Target	           2AT_3	2AT	1	            22.85	TargetB12	Scx	          Target	           2AT_3	2AT	2	            22.76	TargetC12	Scx	          Target   	2AT_3	2AT	3	            22.51	TargetJ13	Gapdh	Control	4AT_1	4AT	1	            22.07	TargetK13	Gapdh	Control	4AT_1	4AT	2	            21.91	TargetL13	Gapdh	Control	4AT_1	4AT	3	            21.87	TargetA13	Scx	          Target	           4AT_1	4AT	1          	 21.85	TargetB13	Scx	          Target   	4AT_1	4AT	2	            21.61	TargetC13	Scx	          Target   	4AT_1	4AT	3	            21.6	TargetJ14	Gapdh	Control	4AT_2	4AT	1	            20.69	TargetK14	Gapdh	Control	4AT_2	4AT	2	            20.92	TargetL14	Gapdh	Control	4AT_2	4AT	3	            20.98	TargetA14	Scx	           Target  	4AT_2	4AT	1	            26.55	TargetB14	Scx	          Target	           4AT_2	4AT	2	            26.47	TargetC14	Scx	           Target 	4AT_2	4AT	3	             26.3	TargetJ15	Gapdh	Control	4AT_3	4AT	1	            18.83	TargetK15	Gapdh	Control	4AT_3	4AT	2	            18.58	TargetL15	Gapdh	Control	4AT_3	4AT	3	            18.96	TargetA15	Scx	           Target  	4AT_3	4AT	1	            23.52	TargetB15	Scx	            Target	4AT_3	4AT	2	            23.51	TargetC15	Scx	            Target	4AT_3	4AT	3	            23.47	TargetJ16	Gapdh	Control	6AT_1	6AT	1	            23.85	TargetK16	Gapdh	Control	6AT_1	6AT	2	           23.52	TargetL16	Gapdh	Control	6AT_1	6AT	3	           23.63	TargetA16	Scx	          Target	           6AT_1	6AT	1	           22.36	TargetB16	Scx	          Target	           6AT_1	6AT	2	           22.23	TargetC16	Scx	          Target   	6AT_1	6AT	3	           22.27	TargetJ17	Gapdh	Control	6AT_2	6AT	1	           22.22	TargetK17	Gapdh	Control	6AT_2	6AT	2	           22	TargetL17	Gapdh	Control	6AT_2	6AT	3	           22.15	TargetA17	Scx	            Target	6AT_2	6AT	1	           26.34	TargetB17	Scx	            Target	6AT_2	6AT	2	          25.96	TargetC17	Scx	            Target	6AT_2	6AT	3	          26.14	TargetJ18	Gapdh	Control	6AT_3	6AT	1         	19.35	TargetK18	Gapdh	Control	6AT_3	6AT	2         	18.82	TargetL18	Gapdh	Control	6AT_3	6AT	3          	18.76	TargetA18	Scx	            Target	6AT_3	6AT	1	          24.73	TargetB18	Scx	            Target	6AT_3	6AT	2	           23.95	TargetC18	Scx	            Target	6AT_3	6AT	3          	24.07	TargetJ19	Gapdh	Control	8AT_1	8AT	1	           24.31	TargetK19	Gapdh	Control	8AT_1	8AT	2	           24.12	TargetL19	Gapdh	Control	8AT_1	8AT	3          	24.21	TargetA19	Scx	            Target	8AT_1	8AT	1          	27.61	TargetB19	Scx	            Target	8AT_1	8AT	2	           27.38	TargetC19	Scx	            Target	8AT_1	8AT	3         	27.55	TargetJ20	Gapdh	Control	8AT_2	8AT	1         	25.8	TargetK20	Gapdh	Control	8AT_2	8AT	2         	25.77	TargetL20	Gapdh	Control	8AT_2	8AT	3         	25.86	TargetA20	Scx	            Target	8AT_2	8AT	1         	28.14	TargetB20	Scx	            Target	8AT_2	8AT	2         	28.09	TargetC20	Scx	            Target	8AT_2	8AT	3         	28.06	TargetJ21	Gapdh	Control	8AT_3	8AT	1         	18.87	TargetK21	Gapdh	Control	8AT_3	8AT	2         	18.85	TargetL21	Gapdh	Control	8AT_3	8AT	3          	18.54	TargetA21	Scx	            Target	8AT_3	8AT	1         	24.26	TargetB21	Scx 	            Target	8AT_3	8AT	2          	23.74	TargetC21	Scx	            Target	8AT_3	8AT	3         	23.85	Target
